# Expression of flavonoid 3’-hydroxylase is controlled by P1, the regulator of 3-deoxyflavonoid biosynthesis in maize

**DOI:** 10.1186/1471-2229-12-196

**Published:** 2012-11-01

**Authors:** Mandeep Sharma, Chenglin Chai, Kengo Morohashi, Erich Grotewold, Maurice E Snook, Surinder Chopra

**Affiliations:** 1 Department of Plant Science, Pennsylvania State University, University Park, Pennsylvania, PA 16802, USA; 2 Center for Applied Plant Sciences and Department of Molecular Genetics, Ohio State University, Columbus, OH, 43210, USA; 3 USDA-ARS, Russell Research Center, 950 College Station Road, Athens, GA, 30605, USA

**Keywords:** Anthocyanins, Flavones, Flavonoids, F3’H, Maysin, Phlobaphenes

## Abstract

**Background:**

The maize (*Zea mays*) *red aleurone1* (*pr1*) encodes a CYP450-dependent flavonoid 3’-hydroxylase (ZmF3’H1) required for the biosynthesis of purple and red anthocyanin pigments. We previously showed that *Zmf3*’*h1* is regulated by C1 (Colorless1) and R1 (Red1) transcription factors. The current study demonstrates that, in addition to its role in anthocyanin biosynthesis, the *Zmf3*’*h1* gene also participates in the biosynthesis of 3-deoxyflavonoids and phlobaphenes that accumulate in maize pericarps, cob glumes, and silks. Biosynthesis of 3-deoxyflavonoids is regulated by P1 (Pericarp color1) and is independent from the action of C1 and R1 transcription factors.

**Results:**

In maize, apiforol and luteoforol are the precursors of condensed phlobaphenes. Maize lines with functional alleles of *pr1* and *p1* (*Pr1*;*P1*) accumulate luteoforol, while null *pr1* lines with a functional or non-functional *p1* allele (*pr1*;*P1* or *pr1*;*p1*) accumulate apiforol. Apiforol lacks a hydroxyl group at the 3’-position of the flavylium B-ring, while luteoforol has this hydroxyl group. Our biochemical analysis of accumulated compounds in different *pr1* genotypes showed that the *pr1* encoded ZmF3’H1 has a role in the conversion of mono-hydroxylated to bi-hydroxylated compounds in the B-ring. Steady state RNA analyses demonstrated that *Zmf3*’*h1* mRNA accumulation requires a functional *p1* allele. Using a combination of EMSA and ChIP experiments, we established that the *Zmf3*’*h1* gene is a direct target of P1. Highlighting the significance of the *Zmf3*’*h1* gene for resistance against biotic stress, we also show here that the *p1* controlled 3-deoxyanthocyanidin and *C*-glycosyl flavone (maysin) defence compounds accumulate at significantly higher levels in *Pr1* silks as compared to *pr1* silks. By virtue of increased maysin synthesis in *Pr1* plants, corn ear worm larvae fed on *Pr1*; *P1* silks showed slower growth as compared to *pr1*; *P1* silks.

**Conclusions:**

Our results show that the *Zmf3*’*h1* gene participates in the biosynthesis of phlobaphenes and agronomically important 3-deoxyflavonoid compounds under the regulatory control of P1.

## Background

The maize (*Zea mays*) flavonoid biosynthesis provides an excellent system to study gene interaction in plants because of its extensive characterization at genetic, biochemical, and molecular levels [1]. Different flavonoid compounds share the same basic skeleton of the flavan-nucleus consisting of two aromatic rings with six carbon atoms (ring A and B) which are interconnected by a hetero-cyclic ring with three carbon atoms (ring C). Maize produces 3-hydroxyflavonoids (anthocyanidins) and 3-deoxyflavonoids which include phlobaphenes, 3-deoxyanthocyanidins, and *C*-glycosyl flavones. These compounds are synthesized in different tissues and this spatial distribution depends on the genetic constitution of the plant. Anthocyanins can accumulate in most plant parts whereas phlobaphenes are predominantly found in kernel pericarp (outer layer of ovary wall), cob-glumes (palea and lemma), tassel glumes, and husk [2]. The 3-deoxyanthocyanins and *C*-glycosyl flavones primarily accumulate in silks [3-5]. However, in some high altitude maize lines *C*-glycosyl flavones can also accumulate in leaves [6] indicating genetic diversity for developmental accumulation of flavonoid metabolites.

The 3-hydroxy- and 3-deoxy-flavonoids in maize are regulated by independent sets of transcription factors. Accumulation of 3-hydroxyflavonoids (anthocyanins) is controlled by two sets of duplicated genes: *colorless1* (*c1*)/*purple leaf1* (*pl1*) are members of the R2R3-MYB family of transcription factors [7], and *booster1* (*b1*)/*red1* (*r1*) are members of the basic helix-loop-helix (bHLH) family [8,9]. Studies have shown that C1 or PL1 proteins interact directly with R1 or B1 to activate transcription of anthocyanin biosynthetic genes in seed and plant body, respectively [10,11]. In contrast, 3-deoxyflavonoid pathway genes are regulated by *pericarp color1* (*p1*), which encodes an R2R3-MYB transcription factor [12]. The *p* locus is a complex of duplicated MYB-homologous genes *p1* and *p2* on chromosome 1 [13]. The *p* locus is a major QTL for the biosynthesis of *C*-glycosyl flavones [14,15] and 3-deoxyanthocyanidins in silks [16].

Three flavonoid biosynthetic genes; *colorless2* (*c2*), *chalcone isomerase1* (*chi1*), and *anthocyaninless1* (*a1*) encode chalcone synthase (CHS), chalcone isomerase (CHI), and dihydroflavonol 4-reductase (DFR), respectively. These three genes are common to the anthocyanin and phlobaphene pathways, but are independently regulated by the corresponding sets of transcription factors [10,17,18]. *In vitro* and *in vivo* studies have shown that C1 + R1 or P1 can direct high level of expression from promoters containing the C1/R1 or P1 binding sites identified previously in the *a1* and *c2* gene promoter [12,19-21].

**Figure 1:**
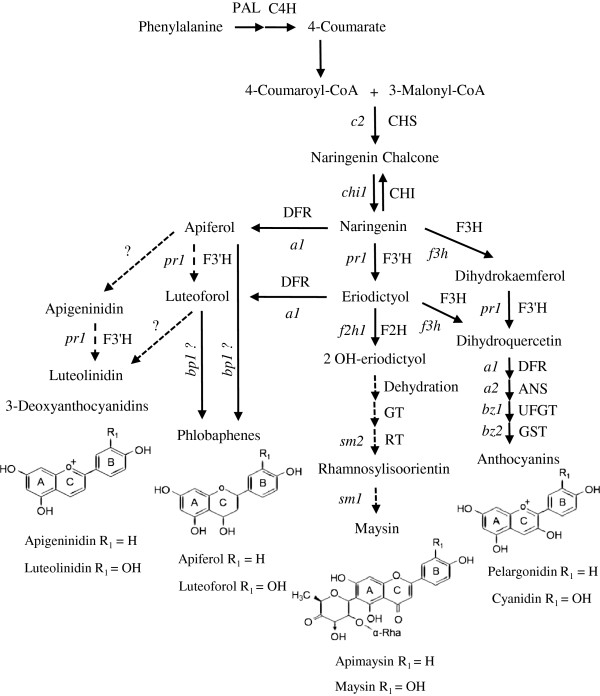
**Flavonoids biosynthetic pathway in maize. **Biosynthetic genes (enzymes) in the pathway are: *c2* (CHS), chalcone synthase; *chi1* (CHI), chalcone isomerase; *f3h* (F3H), flavanone 3-hydroxylase; *pr1* (F3’H), flavonoid 3’-hydroxylase; *a1* (DFR), dihydroflavonol 4-reductase; *a2* (AS), anthocyanidin synthase; *bz1* (UFGT), UDP-glucose flavonoid 3-*O*-glucosyltransferase; and *bz2* (GST), glutathione S-transferase; *f2h1* (F2H), flavanone-2-hydroxylase; GT, *C*-glycosyl transferase; *sm2* (RT), rhamnosyl-transferase; *salmon silk1* (*sm1*) [28]; and *brown pericarp1* (*bp1*) [29,30]; Pathway modeled after [31]; [27] (3-deoxyanthocyanidins); and [21] (C-glycosyl flavones). Putative steps in the pathway are shown as broken arrows with or without probable gene/enzymes involved.

The flavonoid pathway (Figure 1) shows the potential involvement of a flavonoid 3’-hydroxylase (F3’H) in different branches. F3’H is a cytochrome P450-dependent mono-oxygenase and has an influence on the hydroxylation pattern, which is an important structural feature in determining the color and stability of flavonoid compounds [22]. In the anthocyanin branch, F3’H catalyzes the conversion of naringenin to eriodictyol [23]. We have recently demonstrated that the *pr1* gene which encodes a flavonoid 3’-hydroxylase (ZmF3’H1) is required for the accumulation of dihydroquercetin [24]. In addition, a sorghum *f3*’*h* gene has been implicated in different sub-branches of phlobaphene [3], *C*-glycosyl flavone [25,26], and 3-deoxyanthocyanidin pathways [27]. We previously showed that C1 and R1 are required for *Zmf3*’*h1* gene expression in the anthocyanin pathway [24-27]. In the current study we tested the hypothesis of regulation of *Zmf3*’*h1* by P1 in order to explain its role in 3-deoxyflavonoid biosynthesis. Identification of P1 binding sites in the promoter of *Zmf3*’*h1* and *in vivo* P1 binding further established regulation of *Zmf3*’*h1* by P1. Through the genetic and biochemical analysis of well-defined genetic stocks with combination of *pr1* and *p1* alleles we demonstrate that P1 regulated biosynthesis of 3-deoxyflavonoids in pericarps, cob glumes, and silks requires a functional *Zmf3*’*h1* gene.

## Results

### *Pr1*; *P1* cob-glumes accumulate luteoforol

**Figure 2:**
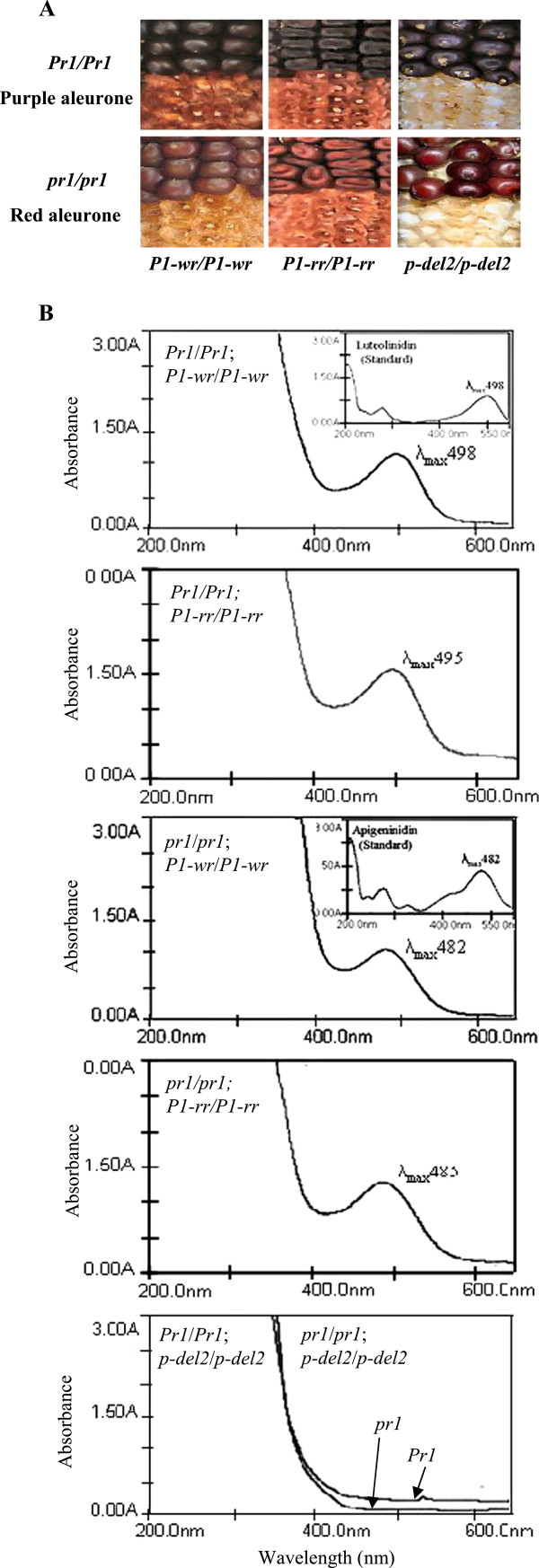
**Luteoforol accumulates in *Pr1; P1 *cob glumes. **(**A**) Ear phenotypes showing cob glumes pigmentation. Cob glumes of *pr1* are light red while *Pr1* has dark red cob glumes in the presence of *P1-wr* or *P1-rr* alleles. *Pr1* and *pr1* ears carrying *p-del2* do not show any pigmentation. (**B**) Absorption spectra of cob glume extracts. Methanolic extracts from cob glumes of *Pr1* ears in genetic background of *P1-wr* and *P1-rr* alleles gave maximum absorption at 498 nm. Cob glumes from *pr1* ears in *P1-wr* and *P1-rr* genetic background gave maximum absorption at 482 nm. These spectra at 498 and 482 correspond to peaks for standard luteolinidin and apigeninidin, respectively (see inset). No absorption peak for flavan 4-ols was observed in *p-del2* cob glumes.

Maize plants carrying a functional *p1* gene accumulate phlobaphene pigments in kernel pericarps and cob glumes. Although, the *Zmf3*’*h1* has been shown to be required for the formation of purple anthocyanins in kernel aleurones [24], changes in pigment intensity has also been observed in phlobaphene accumulating tissues in the presence of a functional *Zmf3*’*h1* gene [3]. To investigate the role of *Zmf3*’*h1* in the *p1* regulated phlobaphene biosynthesis, we developed *Pr1* and *pr1* near isogenic lines in the genetic background of three *p1* alleles: *P1rr*, *P1wr* and *pdel2* (Figure 2A). Phenotypic characterization of *P1wr* ears segregating for *Pr1* and *pr1* showed colorless pericarp and gene dependent cob glumes pigment phenotypes: dark red in *Pr1*/*Pr1*; *P1wr*/*P1wr* while light red in *pr1*/*pr1*; *P1wr*/*P1wr*. Moreover, in the presence of *P1rr*, *Pr1* and *pr1* plants showed pericarp and cob glumes colour differences: *Pr1*/*Pr1*; *P1rr*/*P1rr* plants have dark red pericarp and dark red cob glumes as compared to red pericarp and light red cob glumes in *pr1*/*pr1*; *P1rr*/*P1rr* plants. Importantly, *pdel2* plants (lack both *p1* and *p2*), in the presence of *Pr1* or *pr1* did not show any visible phlobaphene pigmentation in pericarps or cob glumes (see Table 1).

**Table 1:** Genotype and phenotype of different lines developed and used in this study

**Genotype**	**Phenotype**		
	**Aleurone**	**Pericarp**	**Cob-glumes**
*Pr1*/*Pr1*; *P1-rr*/*P1-rr*	Purple	Red	Dark red
*pr1*/*pr1*; *P1-rr*/*P1-rr*	Red	Red	Light red
*Pr1*/*Pr1*; *P1-wr*/*P1-wr*	Purple	Colorless	Dark red
*pr1*/*pr1*; *P1-wr*/*P1-wr*	Red	Colorless	Light red
*Pr1*/*Pr1*; *p1-ww*/*p1-ww*	Purple	Colorless	Colorless
*pr1*/*pr1*; *p1-ww*/*p1-ww*	Red	Colorless	Colorless
*Pr1*/*Pr1*; *p-del2*/*p-del2*	Purple	Colorless	Colorless
*pr1*/*pr1*; *p-del2*/*p-del2*	Red	Colorless	Colorless

Two flavan 4-ols, luteoforol and apiforol have been implicated as precursors of phlobaphene pigments that accumulate in maize genotypes carrying functional *p1* or *p2* genes. Cob glumes were used to perform biochemical characterization of flavan 4-ols. The dark red cob glumes from *Pr1*/*Pr1*; *P1wr*/*P1wr* had maximum absorption (λ max) at 552 nm, while light red cob glumes from *pr1*/*pr1*; *P1wr*/*P1wr* plants had λ max at 535 nm (Figure 2B). These absorption spectra correspond to luteoforol and apiforol, respectively [17]. To further confirm if the *Zmf3*’*h1* plays a role, flavan 4-ols were converted into their corresponding 3-deoxyanthocyanidins by acid treatment of methanolic extracts (Figure 2B). Extracts from *Pr1*/*Pr1*; *P1wr*/*P1wr* converted to luteolinidin (λ max 498 nm) indicating the presence of luteoforol in the methanolic extracts. Similarly, extracts of *pr1*/*pr1*; *P1wr*/*P1wr* cob glumes were converted into apigeninidin (λ max 482 nm) indicating presence of apiforol in the extract. Acid treated methanolic extracts from cob glumes of *Pr1*/*Pr1*; *P1rr*/*P1rr* and *pr1*/*pr1*; *P1rr*/*P1rr* also had maximum absorption wavelengths corresponding to luteolinidin and apigeninidin, respectively. No detectable flavan 4-ols or corresponding 3-deoxyanthocyanidins accumulated in cob glumes of *pr1*/*pr1*; *pdel2*/*pdel2* or *Pr1*/*Pr1*; *pdel2*/*pdel2*. This was in accordance with *p1* gene’s function as a regulator of phlobaphene biosynthesis [12]. Taken together, these results show that cob glumes from *pr1*; *P1* plants accumulate apiforol whereas luteoforol accumulates in cob glumes of *Pr1*; *P1* plants. This result also indicates that the accumulation of apiforol and luteoforol is influenced by a flavonoid 3’-hydroxylase acting in parallel for the conversion of naringenin to eriodictyol.

### *P1* regulates the transcription of *Zmf3*'*h1*

**Figure 3:**
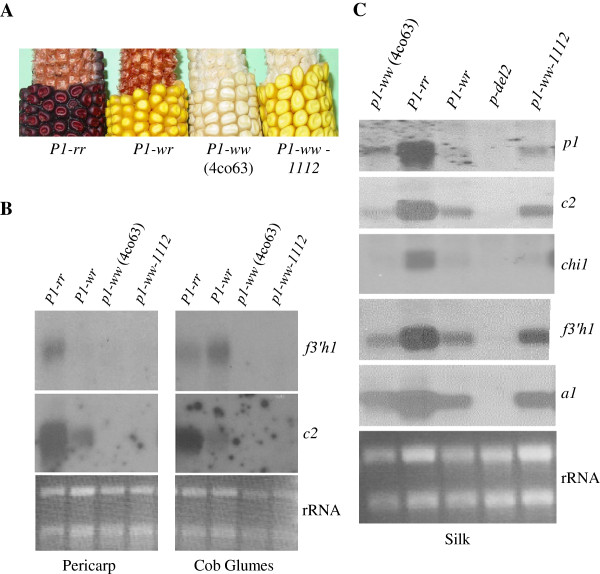
**Expression of *Zmf3’h1 *requires *P1 *and/or *P2 *genes. **(**A**) Ear phenotypes of different *p1* alleles: *P1-rr,* red pericarp, red cob glumes; *P1-wr,* white pericarp, red cob glumes; *P1-ww* [4Co63] and *P1-ww-1112,* white pericarp and white cob glumes. (**B**) Gel blot of RNA extracted from pericarps and cob glumes of different *p1* alleles was probed with *Zmf3’h1* and *c2* probes. *P1-rr* pericarps and cob-glumes and *P1-wr* cob-glumes show *Zmf3’h1* expression. (**C**) RNA gel blot of silks from different *p1* alleles was hybridized with various flavonoid gene fragments as probes. rRNA bands showing equal loading of RNA in different lanes.

The *Zmf3*’*h1* gene appears to affect the composition of flavan 4-ols in floral tissues of *P1* alleles. Thus, it is possible that the *p1* gene regulates the *Zmf3*’*h1* transcription. To test this hypothesis, we examined steady state transcript levels of *Zmf3*’*h1* in pericarps, cob-glumes, and silks of *Pr1* plants carrying functional and null *p1* alleles (Figure 3). Increased levels of *Zmf3*’*h1* transcripts were present in pericarps of *Pr1*/*Pr1*; *P1rr*/*P1rr* plants, while these transcripts were not detected in pericarps of *Pr1*/*Pr1*; *P1wr*/*P1wr* and *Pr1*/*Pr1*; *P1ww*/*P1ww* plants (Figure 3B). In cob glumes of *Pr1*/*Pr1*; *P1rr*/*P1rr* and *Pr1*/*Pr1*; *P1wr*/*P1wr* plants there were appreciable amounts of *Zmf3*’*h1* transcripts, while *Pr1*/*Pr1*; *P1ww*/*P1ww* cob glumes did not show *Zmf3*’*h1* transcripts (Figure 3B). Transcript levels of *p1*, *c2*, *chi1*, *a1*, and *Zmf3*’*h1* were also compared in silks (Figure 3C). Similar to the *p1* expression, *Zmf3*’*h1* was highly expressed in silks of *Pr1*/*Pr1*; *P1rr*/*P1rr* and *Pr1*/*Pr1*; *P1wr*/*P1wr*. Although, both *P1ww* [4Co63] and *P1ww*-1112 alleles have non-functional *p1* gene, detectable levels of *p2* expression was present. The the *p* gene probe used in this study can detect both *p1* and *p2* specific transcripts (see Methods). Thus the *Zmf3*’*h1* expression detected in *P1ww* silks is because of a functional *p2* gene [13]. Further, the *p*-specific transcripts were not detected in *pdel2* silks because of deletions within *p1* and *p2* genes. In addition, other biosynthetic genes *c2*, *chi1*, and *a1* showed expression pattern similar to that of *Zmf3*’*h1* indicating a commonality in the regulation of these genes. Overall, these results show that the *p1* and *p2* genes regulate the transcriptional expression of *Zmf3*’*h1* in maize pericarp, cob glume, and silk.

### P1 binds to two sites in the *Zmf3*’*h1* promoter

**Figure 4:**
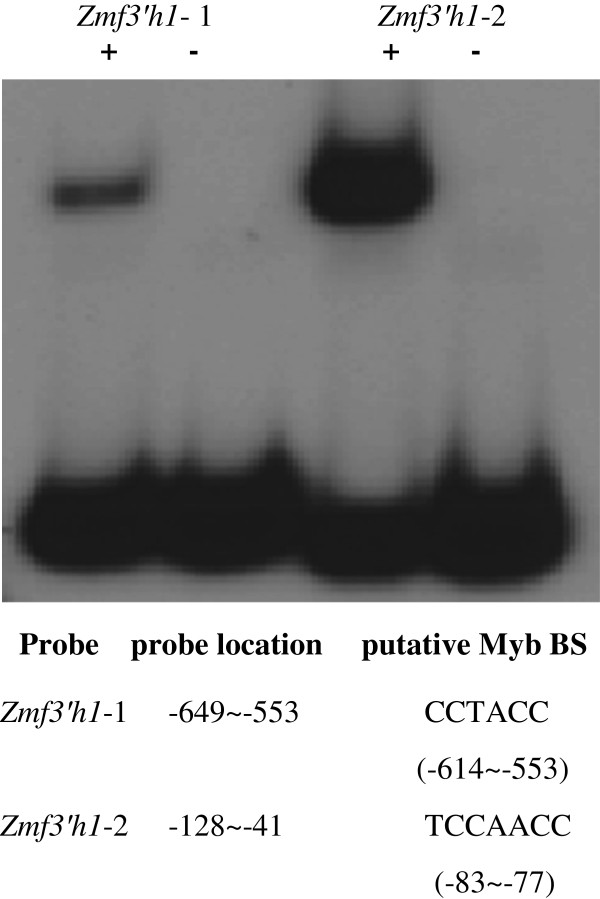
**P1 binds to *Zmf3’h1 *promoter. **The locations of two probes are indicated with reference of the first nucleotide of Transcription Start Site (TSS) as +1. +: P1 + probe; -: free probe.

The *p1* gene regulates expression of *Zmf3*’*h1* during accumulation of luteoforol in maize floral tissues. To further confirm the mechanism by which *p1* controls *Zmf3*’*h1* expression, we found that the *Zmf3*’*h1* promoter contains the *cis*-regulatory elements CCTACC (-614 ~ -553) and CCAACC (-83 ~ -78), that resemble P1 DNA-binding sites [12]. To test this possibility, electrophoretic mobility shift assays (EMSA) were performed using as probes two DNA fragments spanning regions containing the putative P1 binding sites from -649 to -553 and from -128 to -41 (Figure 4). Our results show that P1 binds both the sites, which is in agreement to consensus sequences CC^T^/_A_ACC derived from SELEX [12].

### P1 binds the *Zmf3*^**′**^*h1* promoter *in vivo*

**Figure 5:**
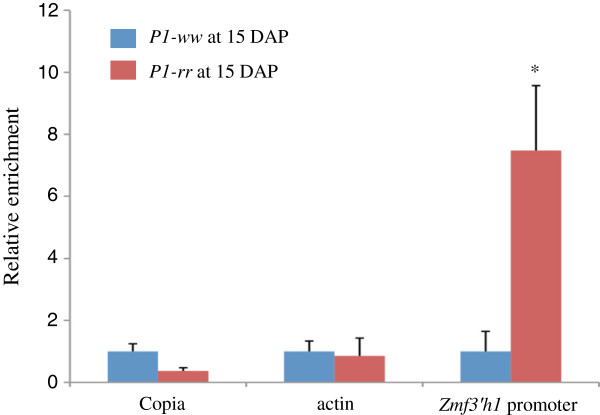
**ChIP assay. **Relative enrichment was measured by a ratio of *P1-rr* to *P1-ww* of % input, which was calculated as a percentage of DNA amount in a fraction of chromatin immuno-precipitation by anti-P1 antibody to total DNA amount in an input fraction. Error bar indicates standard error taken from three independent ChIP experiments. Asterisk shows *P* < 0.05 (*t*-test).

*In vitro* binding assays indicate that P1 directly binds the *Zmf3*’*h1* promoter. In order to investigate whether P1 binds the *Pr1* promoter *in vivo*, chromatin immunoprecipitation (ChIP) experiments were performed. Pericarps from *P1ww* and *P1rr* were collected at 15 days after pollination (DAP), and subjected to ChIP assays using an anti-P1 polyclonal antibody that was successful in *a1* gene ChIP experiments previously [32]. Figure 5 clearly shows that the anti-P1 antibody precipitate complexes containing the *Zmf3*’*h1* promoter fragment with the presence of P1 protein whereas the antibody did not enrich *copia* or *actin* fragments. These results suggest that P1 directly binds *Zmf3*’*h1* promoter *in vitro* and *in vivo*.

### *pr1*; *P1* plants do not accumulate luteolinidin in silks

**Figure 6:**
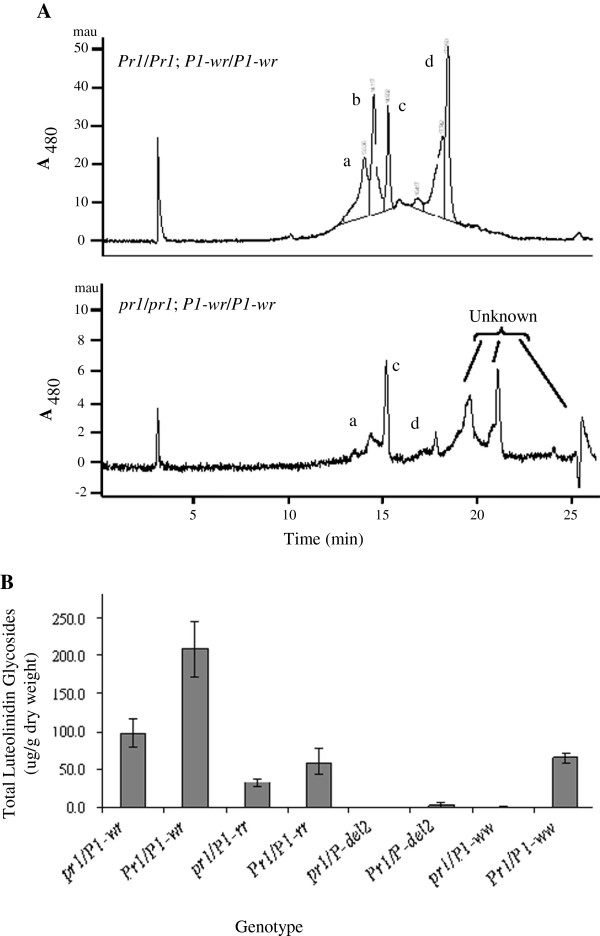
**Characterization of the 3-deoxyanthocyanidins in *Pr1 *and *pr1 *silks. **Compounds were identified based on their retention time to known standards. (**A**) HPLC chromatograms of silk methanolic extracts at 480 nm. Luteolinidin glycosides a, b, and c were eluted at approximately 13.6 min, 14.1 min, and 14.9 min, respectively. Anthocyanidins elute at approximately 17.9 min. (**B**) Total luteolinidin and total anthocyanidin levels in silk tissue of homozygous *pr1*; *P1-wr*, *Pr1*; *P1-wr*, *pr1*; *P1-rr*, *Pr1*; *P1-rr*, *pr1*; *p-del2*, *Pr1*; *p-del2*, *pr1*; *P1-ww*, and *Pr1*; *P1-ww* were determined by HPLC analysis at 495 nm. All data are presented as mean of six replicates.

In maize, *p1* is known to regulate 3-deoxyanthocyanidins in silks, but their biosynthesis is poorly understood [16,17]. Several lines of evidence showed that flavan 4-ols are the precursors of 3-deoxyanthocyanidins [16,17,33-35]. Since, *Zmf3*’*h1* plays a role in the differential accumulation of *p1* regulated flavan 4-ols, we tested if *Zmf3*’*h1* also influences the accumulation of 3-deoxyanthocyanidins. Silk extracts from *Pr1* and *pr1* plants carrying different *p1* alleles were analysed by reverse-phase high performance liquid chromatography (HPLC) (Figure 6). Extracts from *Pr1*/*Pr1*; *P1rr*/*P1rr* and *Pr1*/*Pr1*; *P1wr*/*P1wr* plants showed four major peaks at 480 nm: three for luteolinidin glycosides labelled as a, b, and c with retention times of 13.6 min, 14.1 min, and 14.9 min, respectively and the fourth peak corresponds to anthocyanins, eluting at 17.9 min. (Figure 6A). The peaks a, b, and c had the luteolinidin aglycone spectra but eluted considerably before the aglycone suggesting that they are more polar and therefore most likely correspond to glycosylated flavonoids. Based on their different elution times, these may be mono-glucosyl or di-glucosyl luteolinidins. Interestingly, silks from *pr1*/*pr1*; *P1rr*/*P1rr* and *pr1*/*pr1*; *P1wr*/*P1wr* plants, showed much smaller peaks for all luteolinidin glycosides and few additional peaks of unknown compounds. *Pr1* and *pr1* plants carrying *P1ww* or *pdel2* alleles did not produce any detectable levels of these compounds. This latter result is consistent with the fact that both of these *p* alleles lack a functional *p1* gene to induce 3-deoxyanthocyanidins accumulation in silks. Quantitative HPLC data (Figure 6B) showed that *Pr1*/*Pr1*; *P1rr*/*P1rr* and *Pr1*/*Pr1*; *P1wr*/*P1wr* silks accumulated significantly higher levels of total luteolinidin glycosides as compared to *pr1*/*pr1*; *P1rr*/*P1rr* and *pr1*/*pr1*; *P1wr*/*P1wr*. None or very little amount of 3-deoxyanthocyanidins accumulated in *pdel2* or *P1ww* plants. In summary, HPLC analysis of 3-deoxyanthocyanidins in silks of *Pr1*; *P1*, and *pr1*; *P1* plants show that the *Zmf3*’*h1* participates in the synthesis of luteolinidin.

### Silks of *pr1*; *P1* plants have reduced maysin accumulation

**Figure 7:**
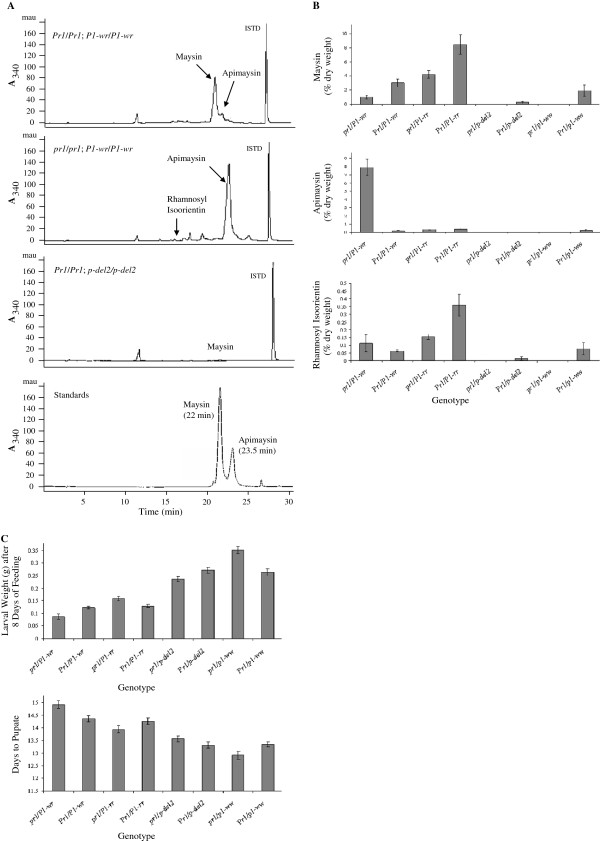
**Characterization of the *C*-glycosyl flavones in *Pr1 *and *pr1 *silks. **(**A**) HPLC chromatograms of silk methanolic extracts from *Pr1* and *pr1* silks measured at 340 nm. Maysin, apimaysin, and rhamnosylisoorientin elute at approximately 22 min, 23.5 min, and 16 min, respectively. (**B**) Maysin, apimaysin, and rhamnosylisoorientin levels in silks of homozygous *pr1*; *P1-wr*, *Pr1*; *P1-wr*, *pr1*; *P1-rr*, *Pr1*; *P1-rr*, *pr1*; *p-del2*, *Pr1*; *p-del2*, *pr1*; *P1-ww*, and *Pr1*; *P1-ww* were determined by HPLC analysis. All data are presented as mean of ten biological replicates. (**C**) Mean corn earworm larval weight and days to pupation on fresh silk from *pr1*; *P1-wr*, *Pr1*; *P1-wr*, *pr1*; *P1-rr*, *Pr1*; *P1-rr*, *pr1*; *p-del2*, *Pr1*; *p-del2*, *pr1*; *P1-ww*, and *Pr1*; *P1-ww* lines.

Our results thus far suggest that, the *Zmf3*’*h1* is under the regulatory control of *p1* and participates in the biosynthesis of both phlobaphenes and 3-deoxyanthocyanidins. To test if *Zmf3*’*h1* also has a role in *p*-regulated *C*-glycosyl flavones biosynthesis, flavone levels in silks of *Pr1* and *pr1* plants in various *p1* allelic backgrounds were measured (Figure 7). Maysin and apimaysin were identified by comparing their retention times of 22.0 min and 23.5 min, respectively and UV absorption spectrum with authentic standards. In *Pr1*/*Pr1*; *P1*-*wr*/*P1*-*wr* silks, maysin was the major compound while smaller peaks for apimaysin and rhamnosylisoorientin were detected at 340 nm (rhamnosylisoorientin retention time was 16.0 min). Silk extracts from *pr1*/*pr1*; *P1*-*wr*/*P1*-*wr* showed a single dominant peak for apimaysin, a smaller peak for rhamnosylisoorientin and no peak for maysin. Peaks for maysin and apimaysin were not observed at an appreciable level in *Pr1*/*Pr1*; *p*-*del2*/*p*-*del2* silks. Quantitative data measured from HPLC showed that *Pr1*/*Pr1*; *P1*-*rr*/*P1*-*rr* and *Pr1*/*Pr1*; *P1*-*wr*/*P1*-*wr* plants have significantly higher levels of maysin and substantially lower levels of apimaysin (Figure 7B, panels I and II). In contrast, *pr1*/*pr1*; *P1*-*wr*/*P1*-*wr* silks produced significantly high levels of apimaysin and very little amount of maysin. In *p*-*del2* silk extracts, no significant levels of *C*-glycosyl flavones were detected irrespective of the type of *pr1* allele present. However, in *Pr1* and *pr1* lines carrying *P1*-*ww* [4Co63] allele, very low levels of maysin and apimaysin were detected; this can be attributed to the functional *p2* gene present in this allele. Interestingly, *Pr1*/*Pr1*; *P1*-*rr*/*P1*-*rr* plants accumulated significantly higher amount of rhamnosylisoorientin in silks as compared to *pr1*/*pr1*; *P1*-*rr*/*P1*-*rr* (see Figure 7B, panel III). Overall, these results suggest that the *Zmf3*’*h1* plays a role in the accumulation of *C*-glycosyl flavones in silks in the presence of functional *p1* alleles.

### *Pr1*; *P1* silks is detrimental to corn earworm larvae development

To determine the biological relevance of differential accumulation of maysin and apimaysin in silks of *Pr1* and *pr1* plants, we performed insect silk feeding bioassays. Corn earworm (*Helicoverpa zea* Boddie) larvae (neonate stage) were fed on fresh silks (Additional file 1: Figure S1) collected from the same *Pr1* and *pr1* plants that were used for HPLC analysis. Larvae fed on *Pr1* silks had lower body weight and took longer time to pupate as compared to those fed on *pr1* silks (Figure 7C). These results are in agreement with the accumulation of higher amounts of maysin in *Pr1* silks. Interestingly, larvae fed on *pr1*/*pr1*; *P1wr*/*P1wr* silks showed lower weight and longer time to pupate as compared to those fed on *Pr1*/*Pr1*; *P1wr*/*P1wr* silks, even though the *Pr1* silks had higher levels of maysin. This anomaly could possibly be because of the accumulation of exceptionally higher level of apimaysin in *pr1*/*pr1*; *P1wr*/*P1wr* (see Figure 7B, panel II). As shown in previous studies, apimaysin has insecticidal activity against lepidopteron insects, although, apimaysin’s activity levels were lower than maysin [36]. Overall, combined data from HPLC and larvae feeding bioassay indicated that a functional *Zmf3*’*h1* participates in the accumulation of 3’-hydroxylated *C*-glycosyl flavones that affect the growth of corn earworm larvae.

## Discussion

The *pr1* locus has been extensively used as phenotypic marker in maize genetics research because of its role in determining kernel aleurone color by hydroxylation of anthocyanin compounds (3-hydroxyflavonoids) [24,37]. However, little is known about the function and regulation of the *pr1* encoded ZmF3’H1 in 3-deoxyflavonoids pathway. The 3-deoxyflavonoids include phlobaphene pigments [38,39] and agronomically important *C*-glycosyl flavones and 3-deoxyanthocyanidins which provide resistance against various biotic stresses [4,31,40-42]. Maize *p1* gene regulates the 3-deoxyflavonoid biosynthetic pathway [12,17]. Here, we describe the first direct evidence of the involvement of *Zmf3*’*h1* in the 3-deoxyflavonoid pathway and its regulation by P1 MYB transcription factor. We have demonstrated that the dark red cob glumes of *Pr1*; *P1* plants accumulates luteoforol as compared to apiforol accumulating in light red cob glumes of *pr1*; *P1* plants. Further, gene expression analysis confirmed that transcription of *Zmf3*’*h1* requires *p1* gene expression in pericarps, cob glumes, and silks. Interestingly, the detection of *Zmf3*’*h1* transcripts in silks of *P1ww* lines suggest that in addition to *p1*, the paralog *p2* is also involved in the regulation of *Zmf3*’*h1* expression in silks. Additionally, the absence of *Zmf3*’*h1* transcripts in *pdel2* allele which has a deletion of *p1* and *p2* genes [14], supported this hypothesis.

P1 is a R2R3-MYB protein and directly regulates the expression of flavonoid biosynthetic genes. Binding of P1 to the *cis*-regulatory elements of the *a1* and *c2* gene promoter has been well characterized [12,21]. Sequence analysis of the *Zmf3*’*h1* promoter shows the presence of similar conserved P1 binding sites. Further, EMSA results demonstrated the *in vitro* binding ability of P1 to these *cis*- sites, while ChIP experiments confirmed that *Zmf3*’*h1* is an immediate direct target of P1. In addition to P1 binding sites, *Zmf3*’*h1* promoter also contains anthocyanin regulatory element (ARE), a conserved sequence present in other anthocyanin biosynthetic genes [24].

Underlining the importance of *Zmf3*’*h1* in maize biotic stress resistance, our work further added that *Pr1*; *P1* plants accumulate significantly higher levels of the antifungal compound luteolinidin in silks. Luteolinidin is known to be toxic towards fungi and it accumulates at higher level in sorghum lines resistant to the anthracnose fungus [43,44]. In sorghum, attempted penetration of *Cochliobolus heterostrophus* leads to up regulation of a *f3*’*h* gene and sequential accumulation of luteolinidin [27]. The 3-deoxyanthocyanidin pathway in sorghum requires a MYB protein encoded by *yellow seed1* (*y1*), an ortholog of maize *p1*[33,45,46]. Similar to the regulation of *Zmf3*’*h1* by *p1*, sorghum *f3*’*h* is regulated by *y1*[46]. It remains to be tested if silk extracts containing higher luteolinidin glycosides show resistance to fungal pathogens of maize.

*Zmf3*’*h1* participates in the biosynthesis of the 3’-hydroxylated *C*-glycosyl flavones with *Pr1*; *P1* silks accumulating higher level of maysin compared to *pr1*; *P1* silks. Structurally, apimaysin and maysin are highly related and differ only by B-ring hydroxylation in position 3’ [47,48]. Unexpectedly, the accumulation of maysin and apimaysin in *Pr1*/*Pr1*; *P1wr*/*P1wr* and *pr1*/*pr1*; *P1wr*/*P1wr* silks did not exactly follow the inverse correlation. Apimaysin level in *pr1*/*pr1*; *P1wr*/*P1wr* silks increased to a substantially higher level as compared to the maysin level in a *Pr1*/*Pr1*; *P1wr*/*P1wr* line. One possibility is that the apimaysin is acting as a substrate for another enzyme and is converted into a product that we were not able to detect in the analysis. We also measured rhamnosylisoorientin which has been shown to have insecticidal activity [49,50]. Rhamnosylisoorientin is a *C*-glycosyl flavone which is present upstream of maysin. Importantly, *Pr1* silks have higher level of rhamnosylisoorientin as compared to *pr1* in the presence of *P1rr* and *P1ww*, respectively. Genetic variation at *p* locus is significantly correlated with maysin accumulation. Genotypes carrying functional *p1* or *p2* alone accumulate less amount of maysin than maize lines that have both *p1* and *p2* genes [51].

Most of the steps in the formation of *C*-glycosyl flavones are unknown. It is possible that formation of *C*-glycosyl flavones does not entirely follow a single linear pathway but demonstrates shunting of substrate flow to alternate pathways through which maysin and apimaysin are formed separately [16,52,53]. The 3’, 4’-hydroxylated flavonoids, such as maysin and luteoforol could also be formed as a result of hydroxylation of naringenin to eriodictyol by F3’H and then subsequent conversion into intermediates leading to formation of these compounds (Figure 1) [28,54]. This could also explain the higher level of rhamnosylisoorientin in *Pr1* plants. A recent study by Morohashi et al (2012) have shown the isolation and cloning of a FNS/F2H encoding gene capable of converting flavanones to 2-hydroxy flavanones, a previously unknown step in the formation of *C*-glycosyl flavones [21]. They have also proposed the formation of 4’ and 3’, 4’- hydroxylated compounds through alternate pathways where a F3’H can perform hydroxylation of naringenin to eriodictyol. The accumulation of different levels of flavones and 3-deoxyanthocyanidins in functional *p1* alleles could be attributed to polymorphic structural genes at different loci: functional *c2*, *whp1*, and *a1* genes have a positive effect on maysin accumulation [16,51].

## Conclusions

The significance of flavonoid defence compounds, 3-deoxyanthocyanidin and *C*-glycosyl flavone has been well established [5,15,55]. The current study attempted to unravel the role of regulatory and biosynthetic genes involved in the synthesis of these flavonoids in order to tailor resistant plants. Through transgenic and non-transgenic studies, it established that functional *p1* and *p2* genes can induce biosynthesis of these compounds [14,17]. The current study along with a previous report [24] demonstrates that *Zmf3*’*h1* plays a significant role in generating diversity in anthocyanin, phlobaphene, 3-deoxyanthocyanidin, and *C*-glycosyl flavone compounds. It will be informative to further analyse the action of *Zmf3*’*h1* at specific steps for the biosynthesis of related phenylpropanoid compounds.

## Methods

### Maize genetic stocks

Standard maize genetic nomenclature is used in the current study [56]. Alleles of the maize (*Zea mays*) *p1* have been identified based on their expression in the floral organs and are named according to their pericarp and cob-glumes pigmentation: *P1wr* (white pericarp, red cob), *P1rr* (red pericarp, red cob), and *P1ww* (white pericarp, white cob) (Figure 3A) [57-60]. The maize inbred lines W23 (genotype *P1wr Pr1 c1 rg*), W22 (*P1wr Pr1 C1 R1*), and other genetic stocks MGS 14273 (*P1wr pr1 C1 R1*) and MGS 14284 (*P1ww pr1 C1 R1*) were kindly provided by the Maize Genetics Co-operation Stock Centre (USDA-ARS, University of Illinois, Urbana, IL). The *P1ww* [4Co63] inbred line was obtained from the National Seed Storage Laboratory (Fort Collins, CO), while *P1rr 4B2*, *P1ww1112* and *pdel2* genetic stocks were obtained from Dr. Thomas Peterson, Iowa State University, Ames, IA [61,62]. The *pdel2* deletion mutant was derived from *P1vv9D9A* and has a deletion encompassing both *p1* and *p2*[14,63]. All *p1* alleles except *pdel2* and *P1ww1112* are in 4Co63 genetic background. Our genetic tests have shown that all these *p* stocks carry a functional *Pr1* allele and their pigmentation phenotypes are presented in Table 1. To develop F_2_ populations, *pr1MGS14273* plants were crossed with *P1wr*, *P1rr4B2*, *P1ww*, and *pdel2* and progenies were grown from selfed F_1_ plants. These F_2_ populations showed a 3:1 segregation for purple to red aleurones. To develop homozygous *Pr1* and *pr1* stocks containing different *p* alleles, plants from F_2_ ears showing desirable pericarp, cob-glumes, and kernel aleurone pigmentation phenotypes (see Table 1) were subjected to six subsequent cycles of self-pollination and selection. To confirm the presence of *Pr1* or *pr1*, PCR based genotyping was done using primers in the promoter region [24].

### Analysis of flavan 4-ols

To detect the presence of flavan 4-ols, 500 mg of cob-glumes were macerated with a plastic grinder in an Eppendorf tube containing 1 mL of 30% HCl/70% butanol (v/v) and incubated for 60 min at 37°C [64]. Samples were spun for 10 min at 14,000 rpm and the absorption spectra of the supernatants were determined using a Shimadzu UV-mini 1240 spectrophotometer (Shimadzu Corporation, Columbia, MD) [65,66]. Apiforol and luteoforol are flavan 4-ols previously described from maize and sorghum that give flavylium ions in acidic butanol with a λ max of 535 and 552 nm, respectively [64]. To confirm the identity of the major flavan 4-ols in cob glumes of *Pr1* and *pr1* alleles in the genetic background of different *p* alleles, methanol extract were treated with aqueous HCl. This converts flavan 4-ols such as apiforol and luteoforol to their corresponding 3-deoxyanthocyanidins (i.e. apigeninidin and luteolinidin). The treated *Pr1* extracts had λ max of 498 nm that shifted in alcoholic AlCl_3_ to a shoulder at 546 nm. The addition of HCl restored its absorption to 498 nm. The results of our samples were verified using commercial standards for apigeninidin and luteolinidin (Extrasynthese, Genay Cedex, France). The commercial sample of apigeninidin had a λ max of 475 nm and did not respond to AlCl_3_, whereas luteolinidin had a λ max of 495 nm that shifted in AlCl_3_ to 546 nm and reverted to 498 nm upon re-addition of HCl.

### RNA gel blot analysis

Silks were collected 2 d after emergence, and pericarps and cob glumes were dissected 20 DAP. To isolate total RNA, tissues were ground in liquid nitrogen and then extracted using Tri-Reagent (Molecular Research Centre Inc., Cincinnati, OH). RNA gel blot hybridizations were performed as described previously [24]. Probe fragments used for RNA gel blot analysis were: plasmid pC2 containing a maize *c2* cDNA [67], pCHI1 containing a maize *chi1* cDNA [68], pA1 with a maize *a1* cDNA [69], pF3’H1 containing *Zmf3*’*h1* cDNA, and pP1 containing full length *p1* cDNA [70]. The *p1* probe used here can recognize both *p1* and *p2* transcripts [71]. Filters were stripped by washing thrice in a boiling solution of 0.1% (w/v) SDS before re-hybridization.

### Protein expression and purification

NHis_6_-P1^MYB^ used for EMSA was expressed in *Escherichia coli* and affinity purified using Ni-NTA beads under natural condition as described previously [72]. Briefly, IPTG induction of 1-liter culture, the cells were harvested by centrifugation and re-suspended in 20 ml of SB buffer (50 mM sodium phosphate, pH 8.0, 100 mM NaCl, and 100 μg/ml phenylmethylsulfonyl fluoride) and passed twice through a French press. The cell lysate was centrifuged and the supernatant was filtered through Mira-cloth (Calbiochem). One ml of 50% slurry Ni-NTA beads (Qiagen) was incubated with the cell lysate supernatant for 2 h with gentle rocking at 4°C. The beads were gently harvested by centrifugation, re-suspended in five ml of SB, and loaded onto a column. The column was washed with SB five times and WB (50 mM sodium phosphate, pH 8.0, 300 mM NaCl, 1% Tween 20, 5 mM 2-mercaptoethanol, 10 mM EDTA, and 10% glycerol) three times. The protein was eluted with five washes of five column volumes of WB containing 50 mM imidazole. The elutions were then dialyzed against A-0 buffer (10 mM Tris pH 7.5, 50 mM NaCl, 1 mM DTT, 1 mM EDTA, and 5% glycerol) and stored at -80°C until further use.

### Electrophoretic mobility shift assay (EMSA)

EMSA was performed as previously described [12]. The two *Zmf3*’*h1* promoter fragments used as probes for EMSA were generated by PCR amplification using the following primer pairs: Pr1-1, 5′- GAGTGGGTTGTGGGATTGTT-3′ and 5′- ACCGTAAGGCCAACTCCAAC-3′; Pr1-2, 5′- GCCCGCGAAGAAAAATATAA-3′ and 5′- CCACTTGCGTGCTTCATCTA-3′, in which one of the primer was radioactively labeled with [γ-^32^P]ATP by using T4-polynucleotide kinase. The radioactively labeled DNA fragments were purified by PAGE and quantified by scintillation counter. Ten ng of purified P1^MYB^ was incubated with an equal molar amount of probes (with radioactivity ~10^5^ CPM) for 30 min and the P1^MYB^-probe complex was separated by PAGE. After PAGE, the gel was dried onto Whatman paper and then subjected to autoradiography at -70°C.

### Chromatin immuno-precipitation assay (ChIP)

ChIP experiments using pericarps were performed as previously described [32]. Real-time PCR was used to detect enriched DNA fragments after ChIP experiments with a minor modification. To adjust for different PCR efficiencies as a consequence of the presence of inhibitory compounds in chromatin obtained from *P1rr* pericarps, equal amounts of the pPHP611 plasmid [73] were spiked in the real-time PCR reaction buffer (Epicentre). Copy number of pPHP611 plasmid [17] in each reaction was estimated by using the normalization primer sets directed to the β-lactamase gene responsible for Amp^r^ in the plasmid. The primer sets used for ChIP-qPCR were the following: qChIP-ZmCopia-F, 5′-CGATGTGAAGACAGCATTCCT-3′, qChIP-ZmCopia-R, 5′-CTCAAGTGACATCCCATGTGT-3′, qChIP-ZmAct1-5UTR-F, 5′-TTTAAGGCTGCTGTACTGCTGTAGA-3′, qChIP-ZmAct1-5UTR-R, 5′-CACTTTCTGCTCATGGTTTAAGG-3′, qrt Zmf3′h1Prom-A1, 5′-AGATCGCGGGTAGGTAGGAG-3′, and qrt Zmf3’h1Prom-B1, 5′-ACTGGTGGCGAGGGTGTAGT-3′. The following primer sets were used to detect Amp^r^ gene: ChIP-Amp-F, 5′-GTAGTTATCTACACGACGGGGAGT-3′, and ChIP-Amp-R, 5′-ATCAGTGAGGCACCTATCTCAGC-3′.

### Analysis of *C*-glycosyl flavones and 3-deoxyanthocyanidins

Primary ear shoots were covered prior to silk emergence to prevent random pollination. Silks were collected on ice 2 d after emergence from the ear and subsequently freeze dried. Silk samples were then shipped on dry ice to the Richard B. Russell Research Centre (USDA-ARS, Athens, Georgia) for biochemical analysis. Flavones were extracted with 125 mL methanol at -20°C for 14 d. Concentration of flavones were determined by reversed-phase HPLC [16,74] and expressed as percent dry silk weight. For 3-deoxyanthocyanidins, silks were extracted for 24 h at -20°C with 10 mL of 1% HCl-Methanol (v/v). Their levels were detected at 495 nm by HPLC, with the same column and solvent program used for flavone analysis. Commercial standard of luteolinidin hydrochloride (different than the luteolinidin standard used for spectrophotometric analysis of cob-glumes) was used for quantification (Roth-Atomergic Chemicals Corp., Farmingdale, N.Y.). Chrysin was used as an internal reference standard. Total 3-deoxyanthocyanidin concentration was calculated as the sum of three distinct luteolinidin glycoside peaks a, b, and c [16].

### Insect bioassay

Corn earworm (*Helicoverpa zea* Boddie) eggs were obtained from Benzon Research Company, Carlisle, PA. Eggs were incubated at 28°C. They hatched after 48 h to produce neonate larvae. Maize lines with *Pr1* or *pr1* alleles in genetic backgrounds of four different *p1* alleles; *P1wr*, *P1rr*, *P1ww*, and *pdel2* were grown during the summer of 2007. Silks collected 2 to 3 d after emergence were pooled from 20 field grown plants per genotype. The experiment was conducted as a randomized complete block design with 30 replications and two cups per replicate. Freshly collected silks were filled into 1 oz. plastic diet cups containing 10 mL of 2.5% (w/v) agar to prevent silk drying. Instead of adding silk extracts to the artificial insect diet, fresh silk tissues were used to maximize the resemblance to natural larval feeding conditions. One neonate larvae was introduced into each cup and larvae were allowed to feed in a controlled environment maintained at 28°C, 75% RH, and a photoperiod of 14/10 h (light/dark). Larval weights were recorded after eight days of feeding and larvae were subsequently transferred to artificial diet [75] until pupation.

## Competing interests

The authors declare that they have no competing interests.

## Authors’ contributions

MS carried out the genetic studies, gene expression analysis, spectrophotometer assays, and insect bioassay, performed the statistical analysis and drafted the manuscript. CC carried out the EMSA assay. KM performed the ChIP analysis. EG participated in the design of the P1 protein interaction with *pr1* promoter study and helped to draft the manuscript. MES carried out the HPLC analysis. SC conceived the study, developed maize genetic stocks, participated in its design and coordination, and helped to draft the manuscript. All authors read and approved the final manuscript.

## Supplementary Material

Additional file 1: **Figure S1. **Corn ear worm silk feeding bioassay. Corn ear worm larvae feeding on silks from *pr1*/*P1*-*wr* (top), *Pr1*/*P1*-*wr* (middle), *Pr1*/*p*-*del2* (bottom) plants. Click here for file
