# Supplementary figures and images for: Expression of flavonoid 3’-hydroxylase is controlled by P1, the regulator of 3-deoxyflavonoid biosynthesis in maize
Source: BMC Plant Biol. 2012 Nov 1;12:196. doi: 10.1186/1471-2229-12-196 (PMC3509002; doi:10.1186/1471-2229-12-196)

Figure S1

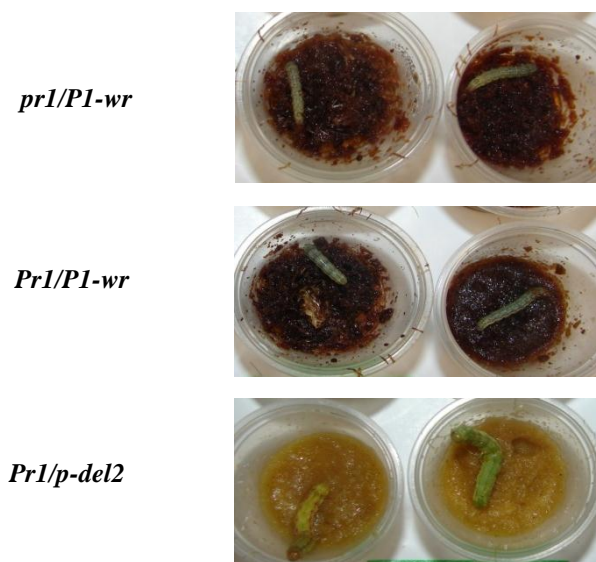

Figure S1. Corn ear worm silk feeding bioassay.

Supplement: Additional file 1: — Figure S1. Corn ear worm silk feeding bioassay. Corn ear worm larvae feeding on silks from pr1/P1-wr (top), Pr1/P1-wr (middle), Pr1/p-del2 (bottom) plants. [file 1471-2229-12-196-S1.pdf]
